# Polyphyllin I Ameliorates Collagen-Induced Arthritis by Suppressing the Inflammation Response in Macrophages Through the NF-κB Pathway

**DOI:** 10.3389/fimmu.2018.02091

**Published:** 2018-09-27

**Authors:** Qiong Wang, Xin Zhou, Yongjian Zhao, Jun Xiao, Yao Lu, Qi Shi, Yongjun Wang, Hongyan Wang, Qianqian Liang

**Affiliations:** ^1^Longhua Hospital, Shanghai University of Traditional Chinese Medicine, Shanghai, China; ^2^State Key Laboratory of Cell Biology, Key Laboratory of Systems Biology, CAS Center for Excellence in Molecular Cell Science, Shanghai Institute of Biochemistry and Cell Biology, Chinese Academy of Sciences, University of Chinese Academy of Sciences, Innovation Center for Cell Signaling Network, Shanghai, China; ^3^Institute of Spine, Shanghai University of Traditional Chinese Medicine, Shanghai, China; ^4^Key Laboratory of Theory and Therapy of Muscles and Bones, Ministry of Education (Shanghai University of Traditional Chinese Medicine), Shanghai, China; ^5^School of Rehabilitation Science, Shanghai University of Traditional Chinese Medicine, Shanghai, China

**Keywords:** polyphyllin I, collagen-induced arthritis, primary macrophages, NF-κB, inflammation

## Abstract

**Background:** Rheumatoid arthritis (RA) is a chronic autoimmune disorder, characterized by an increased number of M1-like macrophages in the joints. Polyphyllin I (PPI), one of the main components in the Rhizoma of Paris polyphyllin, displays a selective inhibitory effect on various tumor cells. Here we sought to investigate the anti-rheumatoid arthritis effects and mechanisms of PPI on macrophages *in vivo* and *in vitro*.

**Materials and Methods:** *In vitro*, primary bone marrow-derived macrophages (BMMs) and peritoneal elucidated macrophages (PEMs) were stimulated by lipopolysaccharide (LPS) and Interferon (IFN)-γ and then treated with PPI. We determined the degree of activation of IKKα/β and p65, two key mediators of the NF-κB-mediated inflammatory pathway, by measuring their phosphorylated forms by Western blot. The p65 nuclear localization was detected by immunofluorescent staining. Further, a NF-κB-linked luciferase reporter plasmid, as well as those expressing key mediators of the Toll-like receptor 4 pathway, such as myeloid differentiation primary response 88 (MYD88), interleukin-1 receptor (IL-1R) associated kinase (IRAK)-1, TNF receptor associated factors (TRAF)-6, Transforming growth factor-b–activated kinase 1 (TAK1) and p65, were used to identify the mechanism by which PPI achieves its inhibitory effects on macrophage-mediated inflammation. Moreover, a NF-κB inhibitor, p65-targeted siRNAs, and a p65 plasmid were further used to validate the anti-inflammatory mechanism of PPI. *In vivo*, PPI (1 mg/kg) was administered intragastrically one time a day for 7 weeks starting on the 42nd day after the first immunization with collagen in a collagen-induced arthritis (CIA) mouse model. Micro-computed Tomography scanning, histological examination, F4/80 and iNOS double immunofluorescent staining and CD4 immunohistochemical staining were performed to determine the effect of PPI treatment on joint structure and inflammation in this model.

**Results:** PPI reduced the inflammatory cytokines production of PEMs stimulated by LPS/IFN-γ, inhibited the phosphorylation of IKKα/β and p65, and prevented p65 nuclear localization. The NF-κB luciferase assay showed that the target of PPI was closely related to the NF-κB pathway. Moreover, NF-κB inhibition, siRNA-mediated knockdown of p65, and p65 overexpression eliminated PPI's inhibitory effect. In addition, PPI attenuated the bone erosion and synovitis, as well as M1-like macrophage and T cell infiltration, in the ankle joint of the CIA model.

**Conclusion:** PPI demonstrated effective amelioration of synovial inflammation in the ankle joint of CIA mice while suppressing NF-κB-mediated production of pro-inflammatory effectors in activated macrophages.

## Introduction

Rheumatoid arthritis (RA) is a chronic autoimmune disease, which is characterized by elevated inflammatory cells infiltration into the synovial joints, eventually resulting in cartilage and bone damage (1, 2). Synovial macrophages are strongly associated with RA severity and are one of the most plentiful cells in the synovial membrane of humans and animals with RA (3, 4). Indeed, disease severity worsens as the number of macrophage increases in the joints, while effective therapy is associated with a decreasing number of macrophages (5, 6). Indeed, it has been reported that macrophages depletion in the joints could reduce the severity of synovial inflammation in a collagen-induced arthritis (CIA) mouse model (7).

Evidence suggests that activated macrophages can critically drive the progression of RA by producing pro-inflammatory cytokines and chemokines, such as interferon-γ (IFN-γ) and tumor necrosis factor-α (TNF-α), which results in the destruction of articular cartilage and sub-chondral bone (8–11). It was reported that the level of IFN-γ is positively correlated with the severity of the disease (12). And TNF-α over-expression in mice leads to RA-like symptoms, while treatment with anti-TNF-α antibodies, such as etanercept and infliximab, can effectively prevent disease progression and protect the joints in the CIA model (13). Collectively, activated macrophages are identified as a treatment target in RA (14).

NF-κB signaling is one of the key transcriptional pathways in RA. In macrophages, the activation of NF-κB in response to Toll-like receptor (TLR) ligands, such as LPS, results in the transactivation of a number of cytokines, chemokines, and other pro-inflammatory mediators (15, 16), and it was reported that TLR4 was activated in RA (16). In the canonical pathway, strong stimuli such as LPS, IFN-γ, and TNF-α can induce the phosphorylation of inhibitor of NF-κB (IκB) kinase (IKK), which mediates the serine phosphorylation and degradation of IκBα, and leads to the release of NF-κB. Liberated NF-κB translocates into the nucleus to regulate the transcription of target genes, including TNF-α, interleukin (IL)-1β, IL-6, and inducible nitric oxide synthase (iNOS) (15–17). Therefore, the blockade of the NF-κB signaling pathway is considered a vital strategy for controlling inflammatory responses in RA (17, 18).

**Figure 1:**
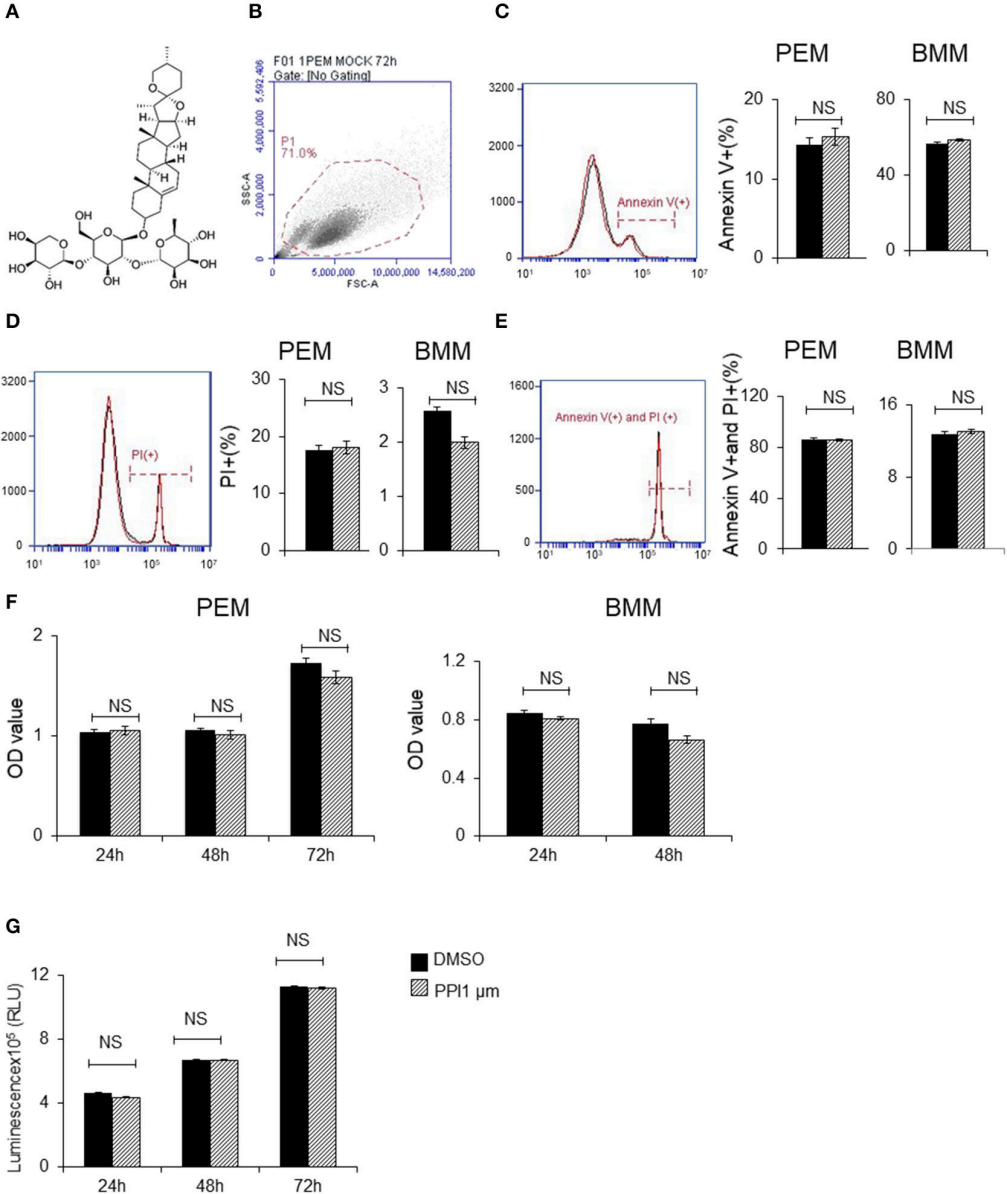
Polyphyllin I does not affect apoptosis or proliferation of macrophages. **(A)** Chemical structure of Polyphyllin I (PPI) (CAS: 50773-41-6). **(B–E)** Cell apoptosis of PEMs and BMMs treated with PPI (1 μM) or the same volume DMSO determined by FITC-conjugated anti-Annexin V antibody and PE-propidium (PI) followed by flow cytometry. Data represent mean ± SEM of three pooled experiments. NS, *P* > 0.05, Student's *t*-test. **(F)** Cell growth of PEMs and BMMs treated with PPI (1 μM) or the same volume DMSO determined by CKK8 assay. Data represent mean ± SEM of four pooled experiments. NS, *P* > 0.05, Student's *t*-test. **(G)** Cell viability of Raw 264.7 cells treated with PPI (1 μM) or the same volume DMSO determined by CellTiter-Glo. Data represent mean ± SEM of four pooled experiments. NS, *P* > 0.05, Student's *t*-test.

Polyphyllin I (PPI, Figure 1A), a small molecular monomer extracted from Rhizoma of Paris polyphyllin, displays a selective inhibitory effect on various tumor cells, including osteosarcoma cells (19, 20), lung cancer cells (21), ovarian cancer cells (22, 23), breast cancer cells (24), and myeloma cells(25). The chemical name of PPI is diosgenyl a-L-rhamnopyranosyl-(1–2)-[b-L-ara-binofuranosyl-(1–4)-b-D-glucopyranoside] with a molecular weight of 855.02 Da, and the chemical formula is C_44_H_70_O_16_ (26). Previously, PPI was reported to down-regulate the constitutive phosphorylation of p65, an NF-κB subunit, in the hepatocellular carcinoma cell line HepG2 (27) and in osteosarcoma cells (20). Those studies demonstrate that PPI has the possibility to affect the NF-κB signaling pathway in several tumor cells. However, whether PPI has therapeutic effects in the CIA mouse model and the relevance to the innate immune system through NF-κB signaling has not been reported previously.

In this study, we sought to investigate the effect of PPI in the CIA mouse model *in vivo* and its mechanism of action in macrophages *in vitro*.

## Methods

### Experimental procedures

#### Mice and ethics statement

We purchased C57BL/6 and DBA/1J mice from Shanghai Slac Laboratory Animal Co. Ltd. We used 6–10 weeks old mice in all experiments. The study protocol was officially approved by the Institutional Animal Care and Use Committee (IACUC) of the Institute of Biochemistry and Cell Biology, Shanghai Institutes for Biological Sciences, Chinese Academy of Sciences (approval numbers 1608-023). All animal procedures were strictly carried out in accordance with the approved guidelines.

### Type II collagen-induced arthritis (CIA) model and PPI treatment

We used a random number table to randomly divide female DBA/1J mice into three groups, including the Mock group, Model + sodium carboxyl methyl cellulose (CMC-Na, which acted as a carrier for PPI) group and Model + PPI group.

Among them, the Mock group consisted of normal mice without collagen injection, while the Model + PPI group and the Model + CMC-Na group represented collagen-induced arthritis (CIA) mice and a carrier control, respectively. Bovine type II collagen (2 mg/ml, Chondrex, cat. # 20021) was fully emulsified with an equal volume of complete Freund Adjuvant (4 mg/ml M. tuberculosis, Chondrex, cat. # 7001). CIA mice were made by subcutaneously injected the emulsion 100 μl at the base of the tail. Generally, the incidence of arthritis was > 90% at 42–56 days.

In the Model + PPI group, PPI (1 mg/kg body weight) was suspended in 0.5% CMC-Na (1 mg/ml) and intragastrically given to CIA mice from day(D) 42 to D91 post first immunization, once a day, while the Mock and Model + CMC-Na group were intragastrically administrated the same volume of CMC-Na as the PPI group. PPI was purchased from the ChemFaces Biochemical Co., Ltd. (cat. # CFN90255).

### Clinical score analysis

Clinical arthritis was evaluated by the following scale: grade *0*, normal (no swelling); grade *1*, mild, but definite redness and swelling of the ankle or wrist, or apparent redness and swelling limited to individual digits, regardless of the number of affected digits; grade *2*, moderate redness and swelling of ankle or wrist; grade *3*, severe redness and swelling of the entire paw including digits; grade *4*, maximally inflamed limb with involvement of multiple joints (28, 29). Each limb was graded with a score of *0* to *4*, with a maximum possible score of 16 for each mouse. Two independent examiners blinded to the treatment group assessed the clinical score once a week.

### Primary cells and cell lines

Primary macrophages, such as bone marrow-derived macrophages (BMMs) and peritoneal elucidated macrophages (PEMs), and two cell lines (Raw 264.7 and 293T) were used in our study. BMMs were obtained from the tibias and femur bones of 8–12 weeks old C57/BL6 mice. After red blood cell lysis, all the cells were seeded into 12-well plates (2 million cells per well) in *macrophage inducing medium* for 5 days with half medium change two times at the third and fourth day. The BMMs were prepared for use after suspending the cells into the *complete 1640 medium* overnight. The *complete 1640 medium* was 1640 medium (Invitrogen, Grand Island, CA, USA) plus 10% (vol/vol) fetal bovine serum (FBS) and penicillin and streptomycin (P/S; 100 U/ml), while the *macrophage inducing medium* is the complete 1640 mediums plus 20 ng/ml murine M-CSF (Peprotech, cat. # 315-02). PEMs were isolated by repeated washing of the abdominal cavity after injecting 3 ml of 3% BBLTM Thioglycollate Medium Brewer Modified medium (BD, REF, USA; cat. # 211716) into 8–12 weeks old C57/BL6 mice in 4 days. 293T and Raw 264.7 cells were obtained from ATCC (ATCC® Number is CRL-11268™ and TIB-71™ respectively). PEMs, 293T, and RAW264.7 were cultured by *completed DMEM medium* consisted of DMEM medium (Invitrogen, Grand Island, CA, USA), 10% (vol/vol) FBS and 100 U/ml P/S.

### siRNA transfection, RNA extraction, and quantitative real-time PCR (qPCR)

Two p65 siRNAs (AGAAGACAUUGAGGUGUAUTT (5′-3′), p65#1 and GAAGAAGAGUCCUUUCAAUTT (5′-3′), p65#2) and the negative control siRNA were transfected into PEMs by Lipofectamine® RNAiMAX Transfection Reagen (cat. # 13778075) strictly under the manufacturer's instructions. Sixty hours after transfection, PPI was added into the medium, 3 h later, LPS/IFN-γ was added into the medium.

Total RNA was prepared by using Trizol (Invitrogen) and the cDNAs were generated by PrimeScript^TM^ RT reagent Kit (cat. # RR047A) according to the manufacturer's instructions. The relative mRNA expression of IL-1β (mouse), IL-6 (mouse), TNF-α (mouse), and NOS2 (mouse), hCCL5 (human), hCXCL10 (human), CD40 (mouse) and CD86 (mouse) were measured by qPCR CFX96 machine (Bio-rad). Hieff^TM^ qPCR SYBR Green Master Mix was purchased from Shanghai Yeasen Biological Technology Co.Ltd. The β-actin acted as a normalization control for all of the mRNAs listed above. The primers for qRT-PCR were shown in Table 1.

**Table 1:** Sequences of Primers Used in the Real-Time Polymerase Chain Reaction.

**Genes**	**Sequences of primers**	**GenBank accession number**	**Annealing Tm(°C)**	**Product size (bp)**
IL-1β	F:5′ CTGGTACATCAGCACCTCAC 3′ R:5′ AGAAACAGTCCAGCCCATAC 3′	NM_008361.4	55	124
IL-6	F: 5′ TGTATGAACAACGATGATGCACTT 3′ R: 5′ ACTCTGGCTTTGTCTTTCTTGTTATCT3′	NM_001314054.1	60	197
TNF-α	F: 5′ AGTGACAAGCCTGTAGCCC 3′: R:5′ GAGGTTGACTTTCTCCTGGTAT 3′	NM_013693.3	57	252
NOS2	F: 5′ GGAGTGACGGCAAACATGACT 3′ R: 5′ TCGATGCACAACTGGGTGAAC 3′	NM_001313922.1	52	127
p65	F: 5′ GATTGAAGAGAAGCGCAAAA 3′ R: 5′ CAGAAGTTGAGTTTCGGGTA 3′	NM_009045.4	55	131
CD40	F: 5′ TGTCATCTGTGAAAAGGTGGTC 3′ R: 5′ ACTGGAGCAGCGGTGTTATG 3′	NM_170702	60	120
CD86	F: 5′ TGTTTCCGTGGAGACGCAAG 3′ R: 5′ TTGAGCCTTTGTAAATGGGCA 3′	NM_170702	61	78
β-actin	F: 5′ CGTTGACATCCGTAAAGACC 3′ R: 5′ TAGGAGCCAGAGCAGTAATC 3′	NM_007393.5	56	110
hCCL5	F: 5′ CCAGCAGTCGTCTTTGTCAC 3′ R: 5′ CTCTGGGTTGGCACACACTT 3′	NM_002985	60	54
hCXCL10	F: 5′ GTGGCATTCAAGGAGTACCTC 3′ R: 5′ TGATGGCCTTCGATTCTGGATT 3′	NM_001565	60	198
hβ-actin	F: 5′ CATGTACGTTGCTATCCAGGC 3′ R: 5′ CTCCTTAATGTCACGCACGAT 3′	NM_001101	60	250

*IL-1β, Mus musculus interleukin-1β; IL-6, Mus musculus interleukin-6; TNF-α, Mus musculus tumor necrosis factor α; NOS2, Mus musculus inducible nitric oxide synthase 2; CD40, Mus musculus CD40 antigen; CD86, Mus musculus CD86 antigen; hCXCL10, Homo sapiens chemokine (C-X-C motif) ligand 10; hCCL5: Homo sapiens chemokine (C-C motif) ligand 5; hβ-actin, homo beta actin. F, forward primer; R, reverse primer*.

### Histology analysis

The ankle joints, kidney and liver were obtained when mice were sacrificed. We soaked the joints in 10% formalin for 48 h after removing the skin and muscle. Then all the left joints were decalcified by a 10% EDTA solution over 21 days. After a series of dehydration, waxing and embedding, the joints were cut into 5 micrometer (μm)-thick slices. The slices were stained by alcian blue/orange G (ABOG) or tartrate-resistant acid phosphatase (TRAP) for histologic analysis.

The samples of kidney and liver tissue were fixed in 10% formalin for 24 h, followed by a series of dehydration, waxing and embedding. And 5 μm sections were cut for hematoxylin and eosin (H&E) staining.

### Immunofluorescence and immunohistochemistry

The slices from ankle joint samples were incubated with anti-F4/80 (Abcam, cat. # ab6640) and anti-iNOS (Abcam, cat. # ab15323) primary antibodies overnight at 4°C. The secondary antibody included Goat-anti-Rat IgG H&L (Alexa Fluor® 647; Abcam. cat. # ab150167) and Goat-Anti-Rabbit IgG H&L (Alexa Fluor® 488; Abcam. cat. # ab150077). And the nuclei were stained by DAPI (4',6-diamidino-2-phenylindole, sigma, cat. # D9564). The images were recorded by an Olympus BX-51 microscope.

For immunofluorescence, PEMs were seeded on round coverslip in 24-well plates. After stimulation at certain time points, cells were fixed by 4% paraformaldehyde for 10 min, permeabilized by 0.5% Triton-X, and blocked by 3% Bovine Serum Albumin (BSA) for 30 min at room temperature. The cells on the coverslip were incubated with anti-NF-κB p65 antibody (Abcam, cat. # ab16502) overnight at 4°C and goat anti-rabbit IgG Alexa Fluor® 488 (Abcam. cat. # ab150077) for 1 h at room temperature. The nuclei were stained by Bisbenzimide H 33342 Trihydrochloride (Hoechst; Sigma Aldrich, CAS: # 23491-52-3). Images were recorded by an Olympus BX-51 microscope.

Immunohistochemistry was performed in accordance with the kit's instructions (Zhongshan Jinqiao PV-9000 Universal Two-Step Test Kit) and the slices were incubated with anti-CD3 antibody (Abcam, cat. # ab56313) overnight at 4°C.

### Serum biochemical parameters determination

Serum aspartate aminotransferase (AST) was tested by *AST reagent kit (Rate method)*, Serum alanine aminotransferase (ALT) was examined by *ALT reagent kit (Rate method)*, Serum creatinine (CRE) was tested by *CRE reagent kit (Enzymatic method)* and blood urea nitrogen (UREA) was detected by *UREA reagent kit (Enzymatic method)*. All the measurements were strictly performed according to the manufacturer's instructions (Shanghai Shensuo UNF medical diagnostic articles Co., LTD).

### Western blot

Cell lysate was prepared by sodium dodecyl sulfate (SDS)-loading buffer lysing and boiled for 10 min, then separated by sodium dodecyl sulfate polyacrylamide gel electrophoresis (SDS-PAGE) and transferred to a Polyvinylidene fluoride (PVDF) membrane. After being blocked in tris-buffered saline and Tween 20 (TBST) with 5% BSA for 1 h at room temperature, the membrane was incubated with primary antibodies, such as Phospho-IKKα (Ser176)/IKKβ (Ser177) Antibody (cat. # 2688), Phospho-NF-κB p65 (Ser536) (93H1) Rabbit mAb (cat. # 3033), Phospho-SAPK/JNK (Thr183/Tyr185) (81E11) Rabbit mAb, (cat. # 4668), Phospho-p44/42 MAPK (Erk1/2) (Thr202/Tyr204) (D13.14.4E) XP® Rabbit mAb (cat. # 4370), Phospho-p38 MAPK (Thr180/Tyr182) (D3F9) XP® Rabbit mAb (cat. # 4511), NF-κB p65 (D14E12) XP® Rabbit mAb (cat. # 8242) or HA-Tag (C29F4) Rabbit mAb (cat. # 3724) overnight at 4°C, and corresponding horseradish peroxidase-labeled secondary antibodies (cat. # 7074), while GAPDH (D16H11) XP® Rabbit mAb (cat. #5174) and β-Actin (8H10D10) Mouse mAb (cat. #3700) coupling horseradish peroxidase itself. All antibodies were purchased from Cell Signaling Technology. Detection was performed with ECL substrate (Thermo Scientific, West Palm Beach, FL, USA). The digital images were recorded by miniature chemiluminescence imaging machine (SAGECREATION, Beijing, China).

### Luciferase assay

pNFκB-luc was purchased from Beyotime Biotechnology (cat. # D2206-1μg). The plasmid was constructed by inserting NF-κB nucleus DNA binding sites into pGL6 plasmid at the multiple cloning sites, which could be used to detect the activation level of NF-κB with high sensitivity. pRL-SV40-C (Beyotime Biotechnology, cat. # D2768-1μg) is a reporter plasmid which can be used to detect the Renilla luciferase reporter gene in mammalian cells.

After transfecting the NF-κB and Renilla luciferase reporters into 293T cells for 8 h by Lipofectamine 2000 reagent (Invitrogen, cat. # 11668-027) according to the manufacturer's instructions (30), we changed the medium with the *complete DMEM medium* containing different doses of PPI (0, 0.25, 0.5, and 1 μM), 3 h later, added human TNF-α (PeproTech, cat. # 300-01A) 20 ng/ml for another 33 h. The double luciferase was detected by the Dual-Luciferase® Reporter Assay System (Promega, cat. # E1910).

3 × HA-tagged human Myd88 (myeloid differentiation primary response 88, NM_001172567), TRAF6 (TNF receptor associated factor 6, NM_145803), IRAK1 (Interleukin 1 receptor associated kinase 1, NM_001569), TAK1 (TGF beta-activated kinase 1, AF218074.1) and p65 (RELA proto-oncogene, NF-kB subunit, NM_021975) was cloned into pcDNA™3.1(+) (Invitrogen, cat. # V79020) at the Multiple Cloning Site. Plasmids expressing Myd88, TRAF6, IRAK1, TAK1, p65, or vector were transfected into 293T cells together with pNFκB-luc and Renilla to measure the relative luciferase reading by PEI (1 μg/μl) (Polysciences, cat. # 23966-2). The double luciferase was also detected by the Dual-Luciferase® Reporter Assay System (Promega, cat. # E1910).

### Th1 and treg differentiation *in vitro*

Pre-coating 48-well plate with anti-CD3 (Bio X cell, cat.# BE0001-1, 1 μg/ml) and anti-CD28 (Bio X cell, cat.# BE0015-5, 1 μg/ml) antibody dissolved into coating buffer (PBS + 10 mM NaHCO_3_, pH = 9.0) at 4°C overnight. Spleen cells from C57/BL6 mice were harvested and seeded as 2 million cells/well in a 48-well plate. Th1 cells (IFN-γ positive) were induced by co-cultured with IL-12 (10 ng/ml) and IL-2 (10 U/ml) for 48 h, while Treg cells (Foxp3-positive) were induced by co-culture with TGF-β (5 ng/ml) and IL-2 (10 U/ml) for 48 h. Then the population was tested by fluorescence activated cell sorting (FACS).

### FACS, enzyme-linked immunosorbent assay (ELISA) and nitric oxide (NO) test

PEMs or BMMs were used for cell apoptosis analysis, while 0.8 × 10^6^ cells were seeded into 12-well plate with 1 ml *completed DMEM medium*. After 12 h of incubation with 1μM PPI, the cells were harvested for staining. Anti-Annexin V (eBioscience, cat: # BMS306APC-20) for flow cytometry were purchased from eBioscience. Propidium iodide (PI) was acquired from Sigma (cat. # P4170). Staining processes were carried out according to the manufacturer's instructions. Briefly, fresh cells were stained with anti-Annexin V-APC antibody for 40 min at 4°C, after washing the cells were suspended with 200 μL FACS buffer (PBS+2%FBS+2‰ sodium azide), then incubated with 0.5 μL PI (100 μg/ml). After 70 μm filter, samples were processed by Accuri C6 (BD Biosciences, San Jose, CA, USA).

For the CD4 and Foxp3 FACS, the cells were stained with FITC Rat Anti-Mouse CD4 (BD Pharmingen™, cat. # 553047) for 40 min at 4°C and then fixed and permeabilized by eBioscience™ Foxp3/Transcription Factor Staining Buffer Set (Invitrogen™, cat. # 00-5523-00). After 40 min of CD4 antibody staining, the cells were fixed by 100 μl 1 × *fix buffer* for 40 min at 4°C, then washed by 1 × *washing buffer*, then incubated by Alexa Fluor® 647 Mouse anti-Human FoxP3 (BD Pharmingen™, cat. # 561184) for 40 min at 4°C. For IFN-γ FACS, the *fix buffer* was 2% paraformaldehyde and the *washing buffer* was FACS buffer.

The ELISA kit of IL-1β, IL-6 and TNF-α were from NeoBioScience and the NO test kit (Griess method) was from Beyotime Biotechnology. To measure IL-1β concentration, SL1344 was added in the supernatant for 15 min to produce mature IL-1β. All test were carried out strictly under the manufactures' instructions.

### Micro-computed tomography (micro-CT) analysis

Right ankle joints were fixed in 10% formalin for 48 h, washed in phosphate-buffered saline (PBS) for 2 h and then soaked in 75% ethanol, scanned by micro-CT system (Scanco VIVA CT80, SCANCO Medical AG, Switzerland). The scanning parameters were as follows: pixel size 15.6 μm, tube voltage 55 kV, tube current 72 μA, integration time 200 ms. The cross-section images were then reconstructed and realigned in 3D, the bone volume (BV) of astragalus were measured and a density threshold was set from 370 to 1000 as *Bone* by μCT Evaluation program V6.6 (Scanco Medical AG, Switzerland). A stack of 340–441 cross-sections was reconstructed, with an inter slice distance of 1 pixel (15.6 μm), corresponding to a reconstructed height of 5.3–6.9 mm, recreating the ankle joints.

### Statistical analysis

Statistical analysis was performed by Graphpad Prism (Version 6.0). Data represent as mean ± standard error of mean (SEM). Statistical significance is determined by unpaired two-tailed Student's *t-*test (or nonparametric test) with 95% confidence intervals for two groups comparison or one-way Analysis of variance (ANOVA) or nonparametric for 3 groups. As to 3 groups with different times points, we applied two-way ANOVA (or nonparametric) comparison. A *P*-value < 0.05 was considered statistically significant.

## Results

### PPI does not show any effect on the apoptosis or proliferation of primary macrophages, or the cell viability of RAW264.7 cells

To avoid the toxic effects of PPI, we firstly examined the effect of PPI on PEMs and BMMs *in vitro*. We found PPI did not affect the early apoptosis or late apoptosis or the death of PEMs or BMMs after incubation for 72 h (Figures 1B–E). In addition, we examined the cell growth activity by CCK-8 kit, and found that PPI did not show any effect on PEMs or BMMs after incubation for 24, 48, or 72 h compared with the equivalent volume of DMSO (Figure 1F). We also investigated the effect of PPI on the cell growth of RAW264.7 cells for 24, 48, and 72 h by Cell Titer-Glo® Luminescent Cell Viability Assay, and obtained similar results that 1 μM PPI did not significantly affect the cell viability of RAW264.7 cells at any time point (Figure 1G).

### PPI reduces the production of pro-inflammatory cytokines by LPS/IFN-γ treated macrophages

**Figure 2:**
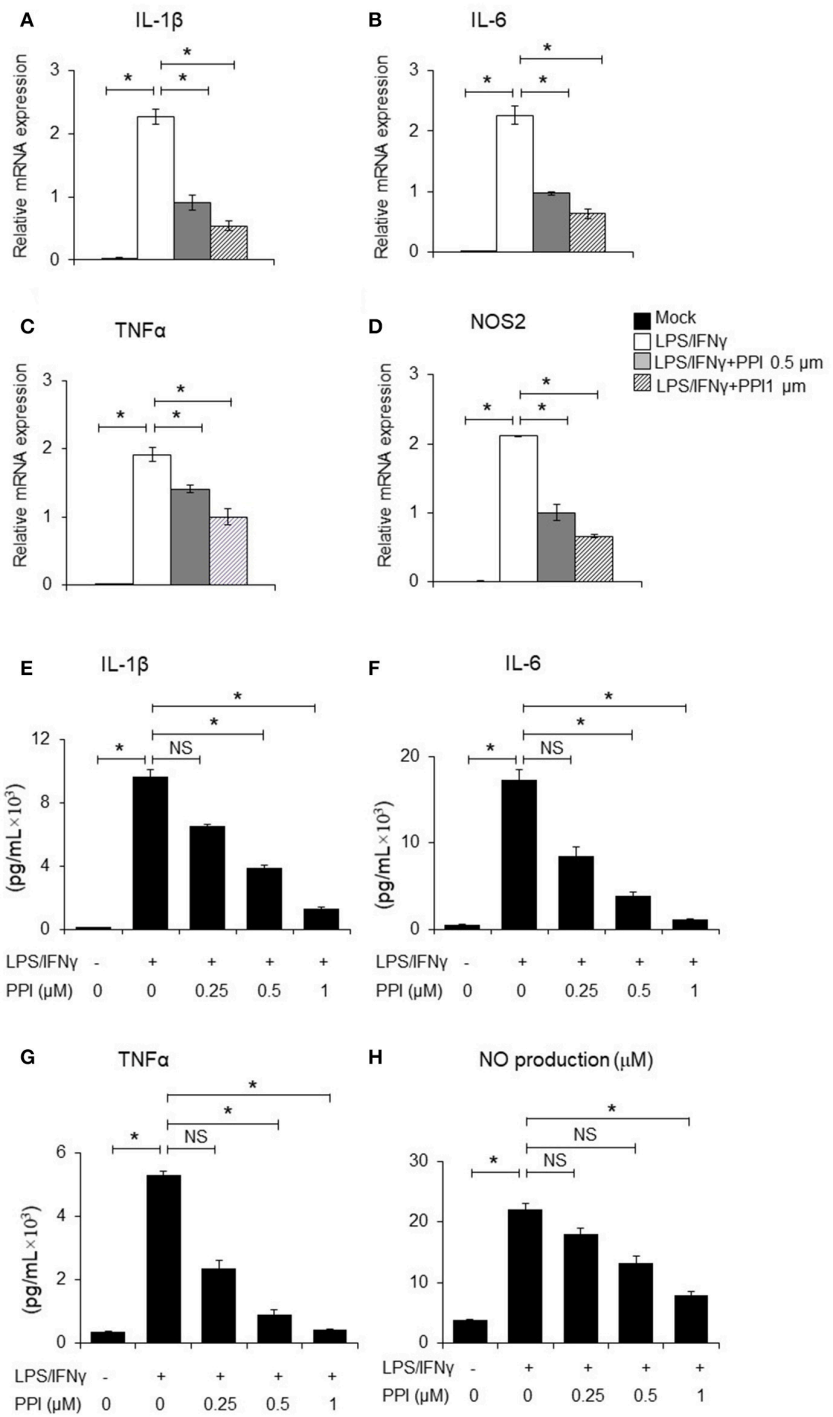
PPI ameliorated the inflammatory cytokines production of BMMs stimulated by LPS/IFN-γ. **(A–D)** IL-1β, IL-6, TNF-α, and NOS2 mRNA expression of BMMs stimulated with LPS (1 μg/ml) /IFN-γ (100 ng/ml) for 6 h in the presence of PPI (0, 0.5, or 1 μM) pretreated for 3 h. Representative data of at least 3 mice. Data represent mean ± SEM. NS, *P* > 0.05, *P < 0.05, One-way analysis of variance (ANOVA). **(E–H)** ELISA in the supernatants of BMMs stimulated with LPS/ IFN-γ for 6 h (IL-1β, IL-6, and TNF-α) and 24 h (NO) in the presence of PPI (0.25, 0.5, or 1 μM) pretreated for 3 h. Data represents mean ± SEM of four pooled experiments. NS, *P* > 0.05, *P < 0.05, One-way ANOVA.

It has been clearly demonstrated that IL-1β and TNF-α, IL-6 are crucial pro-inflammatory cytokines in response to LPS/IFN-γ, while iNOS is a classical pro-inflammatory M1 marker (31–35). BMMs were pretreated with PPI (0.5 and 1 μM) for 3 h, then treated with LPS (1 μg/mL)/IFN-γ (100 ng/ml) for another 6 h. We found that PPI decreased the mRNA expression of IL-1β, TNF-α, IL-6, and iNOS induced by LPS/IFN-γ activated macrophages in a concentration-dependent manner (Figures 2A–D). Furthermore, we also tested the concentration of IL-1β, TNF-α, and IL-6 in the supernatant of BMMs by ELISA after the pretreatment of PPI (0.25, 0.5, and 1 μM) for 3 h and LPS/IFN-γ treatment for another 6 h. We also investigated nitric oxide (NO) production in the conditioned medium of BMMs after 3 h of PPI pretreatment (0.25, 0.5, and 1μM) following LPS/IFN-γ treatment for 24 h. We found that PPI significantly decreased the production of IL-1β, TNF-α, IL-6, and NO by BMMs in a concentration-dependent manner (Figures 2E–H).

### PPI inhibits the phosphorylation of IKKα/β and p65, and p65 nuclear accumulation, without any effect on MAPK signaling

**Figure 3:**
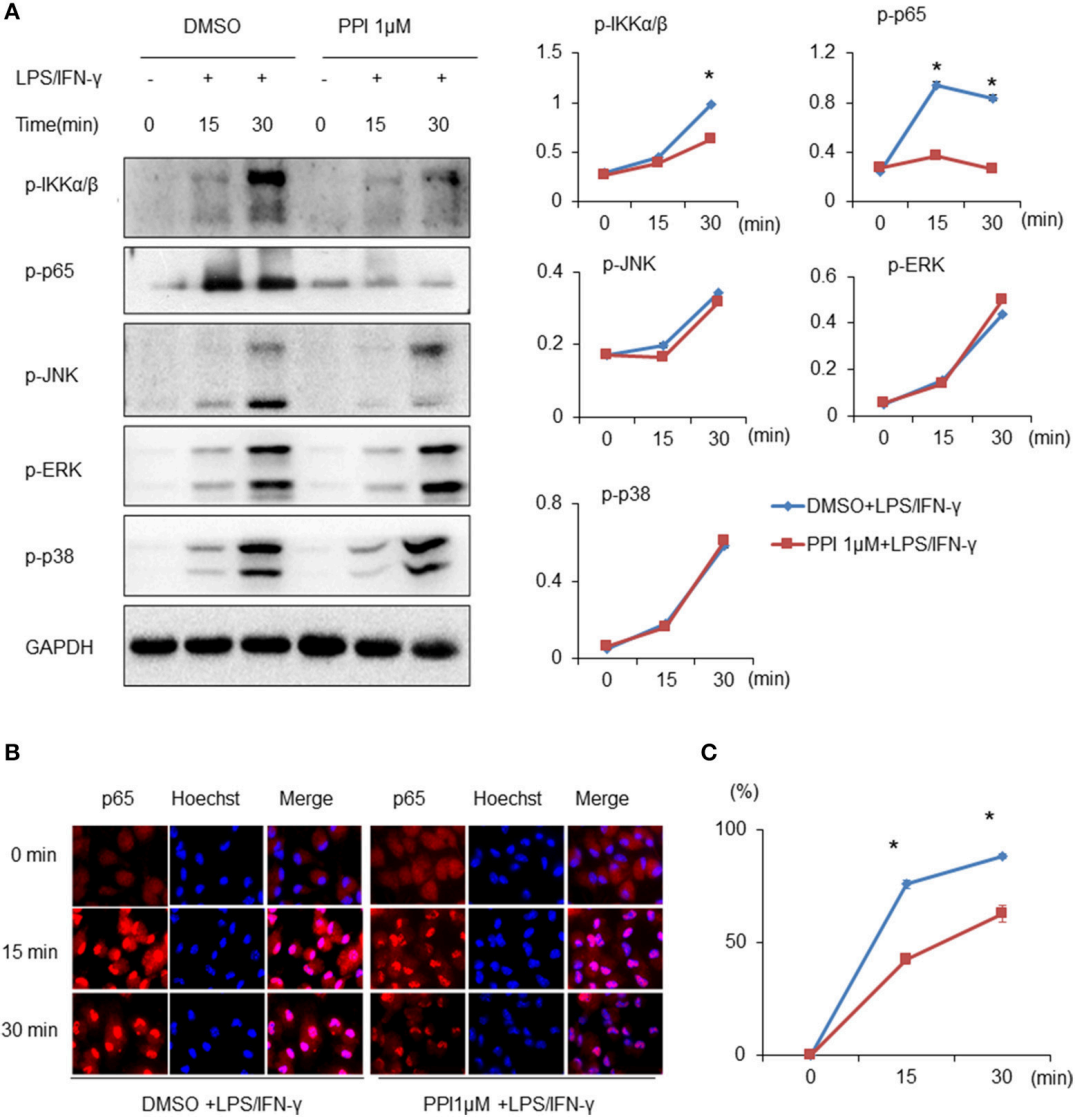
PPI ameliorated the phosphorylation of IKKα/β and p65, and p65 nuclear accumulation, without any effect on MAPK signaling under the stimulation of LPS/IFN-γ. **(A)** Immunoblots for phosphorylation of IKKα/β, p65, JNK, ERK, p38, and the protein level of GAPDH in cell lysates of BMMs stimulated with LPS (1 μg/ml)/IFN-γ (100 ng/ml) for 15, 30, and 60 min in the presence of PPI (1μM) or the same volume DMSO pretreated for 3 h. Blot is representative of three independent experiments. The normalized density of was shown on the right. Data represent mean ± SEM of three pooled experiments. *P < 0.05, two-way ANOVA. **(B, C)** PEMs were unstimulated or stimulated with LPS (1 μg/ml) /IFN-γ (100 ng/ml) for 15, 30, and 60 min in the presence or absence of PPI pretreatment for 3 h. The cells were stained with anti-p65 (in red) and Hoechst (in blue). Representative 100 × images. More than 300 cells from each sample were measured. The values are the mean ± SEM. The experiment was repeated at least three times. *P < 0.05, two-way ANOVA.

Both NF-κB and MAPK (JNK 1/2, p38 MAPK, and ERK 1/2) are critical downstream mediators of TLR signaling and participate in regulating pro-inflammatory mediators and cytokines production (36–38). Thus, we examined whether PPI regulates the activation of MAPKs or NF-κB signaling by LPS/IFN-γ-stimulated macrophages. We found that the phosphorylation of IKKα/β, p65, JNK 1/2, p38 MAPK, and ERK 1/2 of BMMs were markedly increased after LPS/IFN-γ stimulation at 15 and 30 min. Treatment of PPI (1 μM) significantly inhibited LPS/IFN-γ-induced phosphorylation of IKKα/β and p65, without any effect on the phosphorylation of JNK 1/2, p38 MAPK and ERK 1/2 in BMMs (Figure 3A). In addition, an immunofluorescence assay revealed that PPI significantly reduced the level of p65 in the nucleus of PEMs induced by LPS/IFN-γ (Figure 3B).

### PPI reduces NF-κB luciferase activity in a 293T cells

**Figure 4:**
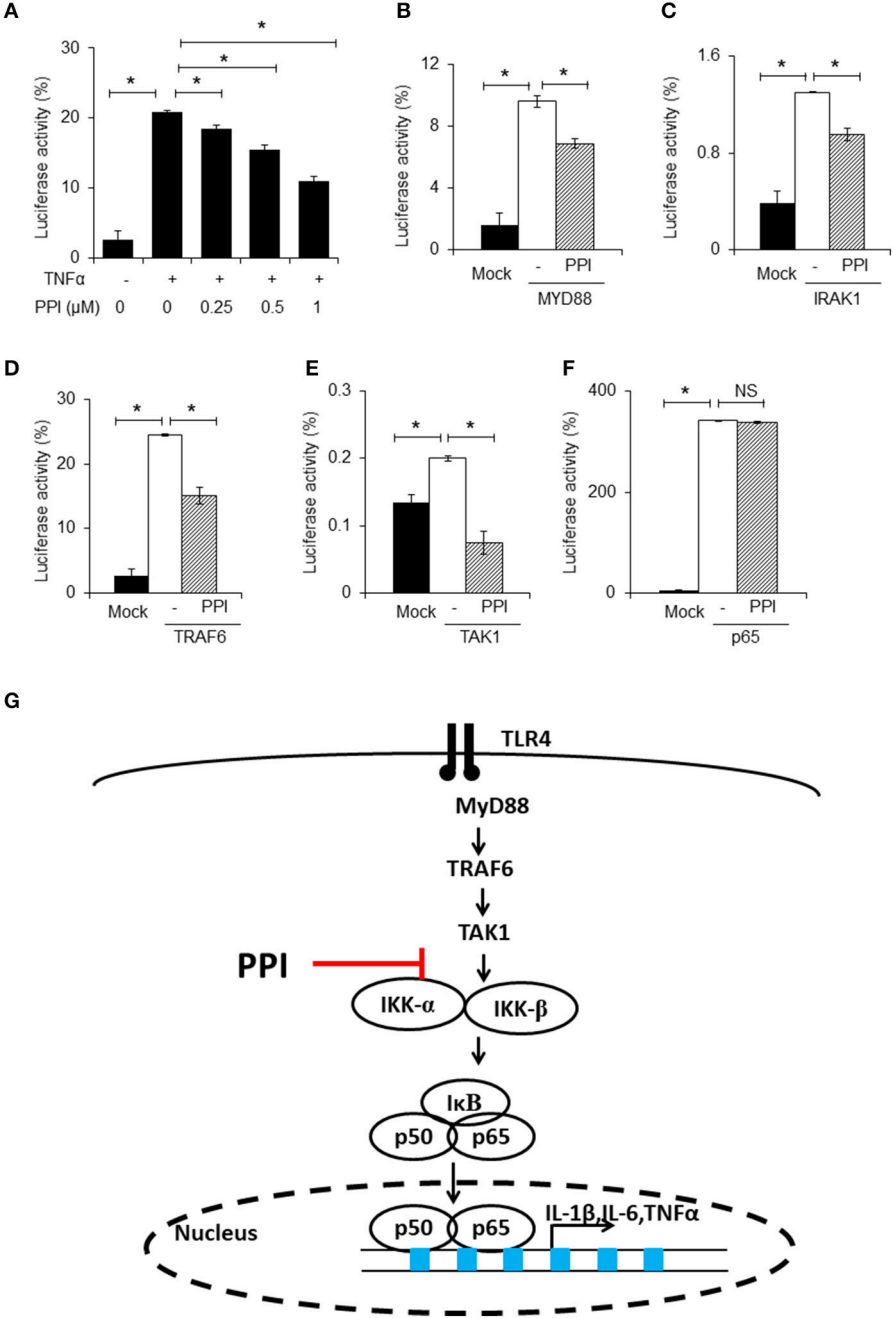
PPI inhibited NF-κB luciferase activity. **(A)** NF-κB and Renilla luciferase plasmid were co-transfected into 293T cells. 8 h later, the cells were incubated with different doses of PPI (0, 0.25, 0.5, 1 μM) for 3 h, then treated with human TNF-α (20 ng/ml) for another 15 h, and the NF-κB and Renilla luciferase was detected by using Dual Luciferase® Reporter Gene Aassy Kit. Data represent mean ± SEM of three pooled experiments. *P < 0.05, one-way ANOVA. **(B–F)** The 293T cells, after transfection with MYD88, IRAK1, TRAF6, TAK1 or vector plasmids together with the NF-κB luciferase and Renilla plasmid for 8 h, was cultured with PPI (1 μM) for another 24 h, and the double luciferase was detected. Data represent mean ± SEM of three pooled experiments. NS, *P* > 0.05, *P < 0.05, one-way ANOVA. **(G)** Diagram illustrating the inhibitory effect of PPI on the TLR4 pathway.

To further confirm the effect of PPI on NF-κB signaling, we used an NF-κB luciferase gene reporter assay. Briefly, after transfection with NF-κB luciferase and Renilla plasmids into 293T cells for 6 h, PPI (0, 0.25, 0.5, 1 μM) was added into the culture medium for 3 h, and then the human TNF-α (20ng/ml) was added for another 15 h. The NF-κB luciferase activity was detected by the double luciferase system. We found that PPI diminished TNF-α-stimulated NF-κB luciferase activation in a dose-dependent manner (Figure 4A). Since there are no TLRs on 293T cells, we used human TNF-α to mimic the activation of NF-κB in 293T cells.

In the next step, we separately transfected MYD88, IRAK1, TRAF6, TAK1, or p65 expression plasmids into 293T cells, which were transfected with NF-κB luciferase and Renilla plasmids at the same time, to mimic the activated state of the NF-κB pathway. We found that PPI could inhibit NF-κB transcription when MYD88, IRAK1, TRAF6, and TAK1 were overexpressed, but could not block NF-κB activation when p65 was overexpressed (Figures 4B–F). The findings are summarized in Figure 4G.

### An NF-κB inhibitor, p65 siRNAs and p65 overexpression block the inhibitory effect of PPI on LPS/IFN-γ-induced inflammatory cytokines expression of BMMS

**Figure 5:**
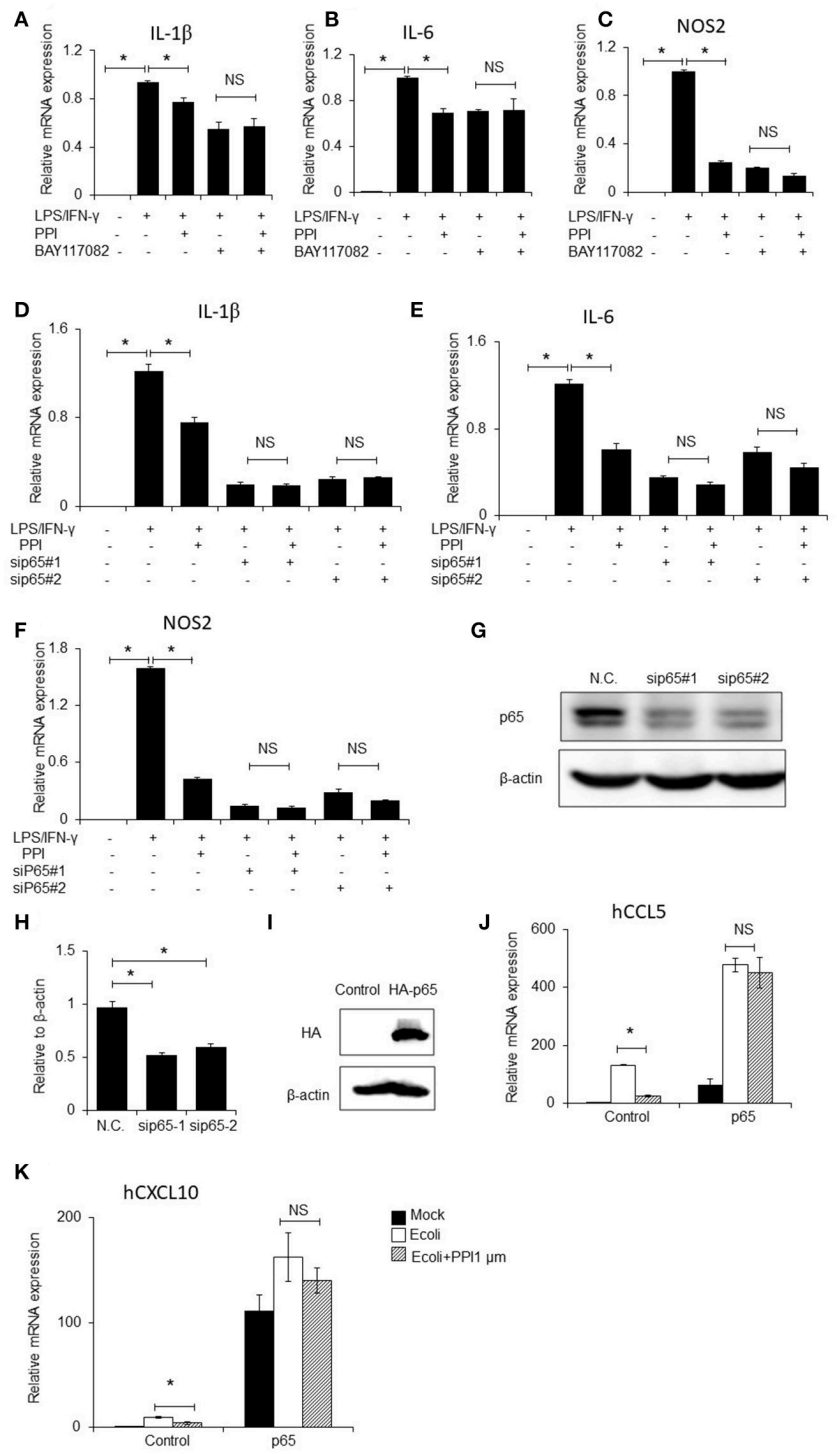
NF-κB inhibitor, p65 siRNAs and overexpression of p65 abolishes the inhibitory effect of PPI. **(A–C)** IL-1β, IL-6, and NOS2 mRNA expression of BMMs were treated with the NF-κB inhibitor BAY117082 (5μM) and PPI (1 μM) for 3 h and LPS/IFN-γ for another 6 h. Data represent mean ± SEM of three pooled experiments. NS, *P* > 0.05, *P < 0.05, one-way ANOVA. **(D–F)** IL-1β, IL-6, and NOS2 mRNA expression of PEMs transfected with siP65#1 and siP65#2 for 60 h, then with PPI (1 μM) or the same volume DMSO for 3h, LPS/IFN-γ for another 6 h. Data represent mean ± SEM of three pooled experiments. NS, *P* > 0.05, *P < 0.05, one-way ANOVA. **(G)** Immunoblots for p65 of PEMs transfected with siP65 for 60 h. Blot is representative of three independent experiments. **(H)** The density of p65 was normalized against β-actin. Data represent mean ± SEM of three pooled experiments, *P < 0.05, one-way ANOVA. **(I–K)** 293T cells were transfected with p65 plasmid for 36h, following PPI (1μM) for 3h and *E.coli* (5 × 10^7^ CFU/ml) for another 6 h, immunoblots for p65, and CCL5, and CXCL10 mRNA expression were determined by qPCR. Blot is representative of three independent experiments. Data represent mean ± SEM of three pooled experiments. *P < 0.05, NS, *P* > 0.05, one-way ANOVA.

To further investigate whether NF-κB signaling contributes to PPI-inhibited inflammatory cytokines expression, we firstly blocked NF-κB signaling by treating BMMs with the NF-κB inhibitor BAY117082 (Sigma, CAS:19542-67-7, 5 μM), followed by treatment with PPI. We then treated the cells with LPS/IFN-γ and measured the mRNA expression of IL-1β, IL-6 and iNOS. We found that NF-κB inhibition decreased LPS/IFN-γ-induced inflammatory cytokines expression, and PPI did not significantly inhibit LPS/IFN-γ-induced inflammatory cytokines expression in the presence of the NF-κB inhibitor (Figures 5A–C).

We next developed two p65 siRNAs, and transfected them into PEMs, followed by treatment with PPI and LPS/IFN-γ. qPCR results indicated that PPI significantly reduced LPS/IFN-γ-induced IL-1β, IL-6, and iNOS mRNA expression in the treated PEMs, but PPI could not significantly inhibit LPS/IFN-γ induced inflammatory cytokines expression when the cells were transfected with the two p65 siRNAs (Figures 5D–F). The ability of the two siRNAs to knock down p65 expression was confirmed by Western blot (Figures 5G, H). In addition, we transfected p65-3 × HA-pcDNA3.1 or the vector plasmid into 293T cells for 36 h, added PPI for another 3 h then stimulated 293T by *E.coli* (5 × 10^7^ CFU/ml) for another 6 h, and found that PPI could not inhibit the expression of CCL5 or CXCL10 mRNA expression induced by *E.coli* when p65 was overexpressed (Figures 5I–K).

### PPI does not affect CD40 and CD86 expression triggered by LPS/IFN-γ

**Figure 6:**
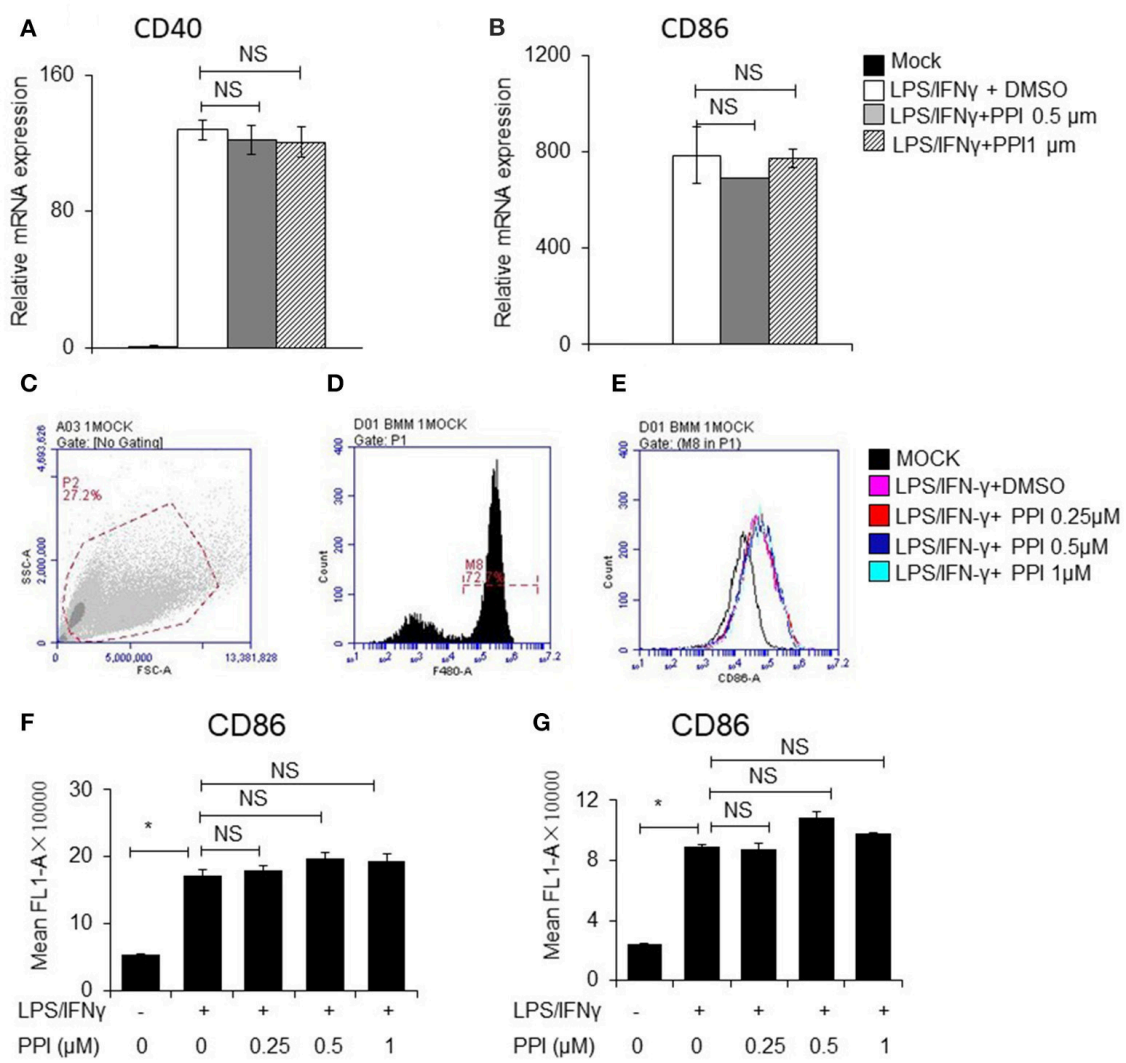
PPI did not affect CD40 and CD86 expression triggered by LPS/IFN-γ, which does not require NFκB activation. **(A, B)** CD40 and CD86 mRNA expression of BMMs was pretreated with PPI (0.25, 0.5, or 1 μM) or the same volume DMSO for 3 h, then stimulated with LPS (1 μg/ml) /IFN-γ (100 ng/ml) for another 6 h. Data represent mean ± SEM. NS, *P* > 0.05, one-way ANOVA. **(C–G)** BMMs or PEMs were pretreated with PPI (0.25, 0.5, or 1 μM) or the same volume DMSO for 3 h, then treated with LPS (1μg/ml)/IFN-γ (100ng/ml) for another 24 h, CD86 level were examined by flow cytometry. Data represent mean ± SEM. NS, *P* > 0.05, *P < 0.05, one-way ANOVA.

In order to determine whether PPI can affect other signaling pathways stimulated by LPS/IFN-γ, we examined the mRNA and protein expression of CD40 and CD86, which are triggered by LPS/IFN-γ but does not require NF-κB activation. We pretreated PEMs or BMMs with PPI (0.5 or 1 μM) for 3 h, then LPS/IFN-γ for another 6 h, and found the mRNA expression of CD40 and CD86 increased under the stimulation of LPS/IFN-γ, while the up-regulation was not significantly reduced at any concentration of PPI (Figures 6A, B). We also investigated the protein expression of CD86 by FACS after PPI pretreatment for 3 h and LPS/IFN-γ treatment for another 24 h, and found that PPI could not decrease CD86 protein level under the stimulation of LPS/IFN-γ (Figures 6C–G).

### PPI attenuates the severity of synovial inflammation and macrophages infiltration in the ankle joint of CIA mice

**Figure 7:**
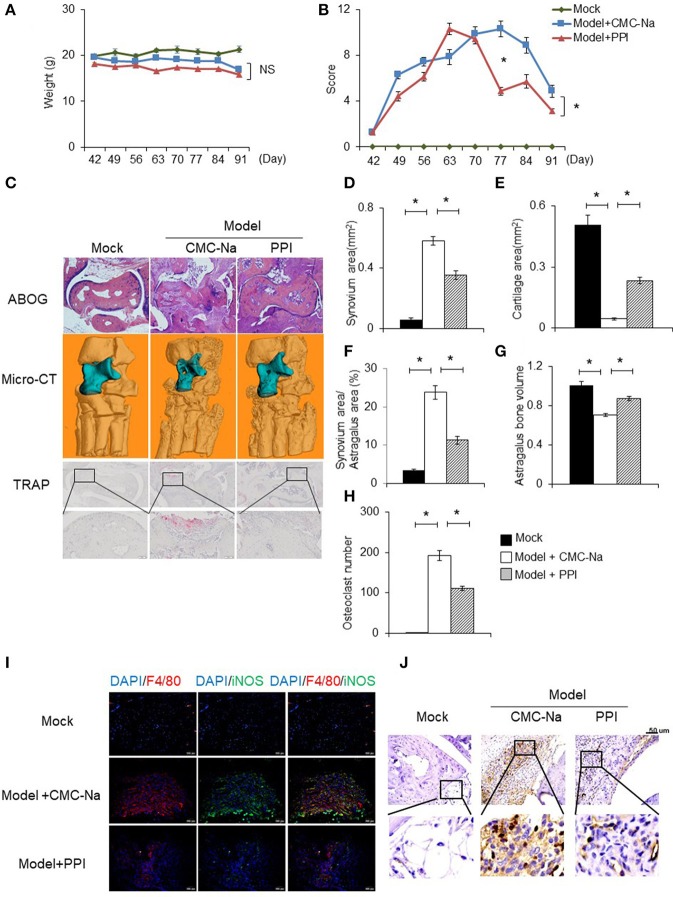
PPI attenuated collagen induced rheumatoid arthritis and protected bone erosion. CIA mice were treated intragastrically with CMC-Na with or without PPI (10 μg/kg) daily from day 42 after immunization to day 91. All mice were sacrificed on day 91. **(A, B)** The weight and arthritis swelling severity scores in CIA mice were recorded weekly from day 42 to day 91 after immunization. Data represent mean ± SEM of two pooled experiments, *n* = 4 for Mock, *n* = 7 for Model+CMC-Na and *n* = 7 for Model+PPI group. NS, *P* > 0.05, *P < 0.05, two-way ANOVA. **(C)** Representive images of ABOG-staining (4×), micro computed tomography three–dimensional and TRAP staining images (4×) of ankle joints. In micro computed tomography three–dimensional ankle joints, the blue part indicates astragalus, *n* = 4 for Mock, *n* = 7 for Model+CMC-Na and *n* = 6 for Model+PPI group. **(D–H)** Synovial area in the ankle joint, the cartilage area of the astragalus, the synovial area/astragalus area and astragalus bone volume, and osteoclasts number of the joint were calculated. Data represent mean ± SEM of two pooled experiments, *n* = 4 for Mock, *n* = 7 for Model+CMC-Na and 6 for Model+PPI group. *P < 0.05, one-way ANOVA. **(I)** Double immunofluorescence staining with anti-F4/80 (red) and anti-iNOS (green) antibodies at ankle sections show that decreased activated macrophages (double positive of F4/80 and iNOS) in the synovium of PPI-treated ankle joints, representative images (20×) from the Mock group (*n* = 4), the Model+CMC-Na (*n* = 7) and the Model+PPI group (*n* = 6). **(J)** Representative images of immunohistochemical staining with anti-CD3 antibody in the synovium around astragalus, bar indicates 50 μm, *n* = 4 for Mock, *n* = 7 for Model+CMC-Na and *n* = 6 for Model+PPI group.

To determine the *in vivo* effect of PPI on RA, we treated CIA mice with PPI. Because PPI is insoluble in water, we utilized CMC-Na to suspend PPI, and used this vehicle alone as a negative control. We found that PPI treatment did not significantly cause weight loss in CIA mice (Figure 7A). Evaluation of clinically relevant endpoints showed that the severity score in the PPI-treated group was significantly less than the control group at day 77, when the score reached a peak in the control group (Model + CMC-Na; Figure 7B). Histological examination and histomorphometric analysis showed that ankle joints from the Model + CMC-Na group had severe inflammation and extensive cartilage and bone damage, and the inflammatory synovium area was significantly reduced in ankles of PPI-treated mice (Figures 7C–F). Three-dimensional micro-CT revealed that the bone volume of astragalus was significantly reduced in the Model + CMC-Na group, and the bone volume of talus in the Model +PPI group was significantly greater than the Model + CMC-Na group (Figures 7C, G). TRAP staining showed that the Model +PPI group had fewer osteoclasts around the astragalus in the ankle joints than the Model + CMC-Na group (Figures 7C, H).

To confirm the *in vivo* anti-inflammatory effect of PPI in CIA mice was through inhibition of the inflammatory response of macrophages, double immunofluorescence (IF) staining on ankle tissue sections was performed with labeled anti-bodies against the M1-specific antigen F4/80 and iNOS (39–44). Both positive staining of F4/80 and iNOS indicate an inflammatory response was activated at macrophages. The results demonstrated that abundant double-positive macrophages existed in the ankle joint of CIA mice, while PPI significantly reduced their presence (Figure 7I).

**Figure 8:**
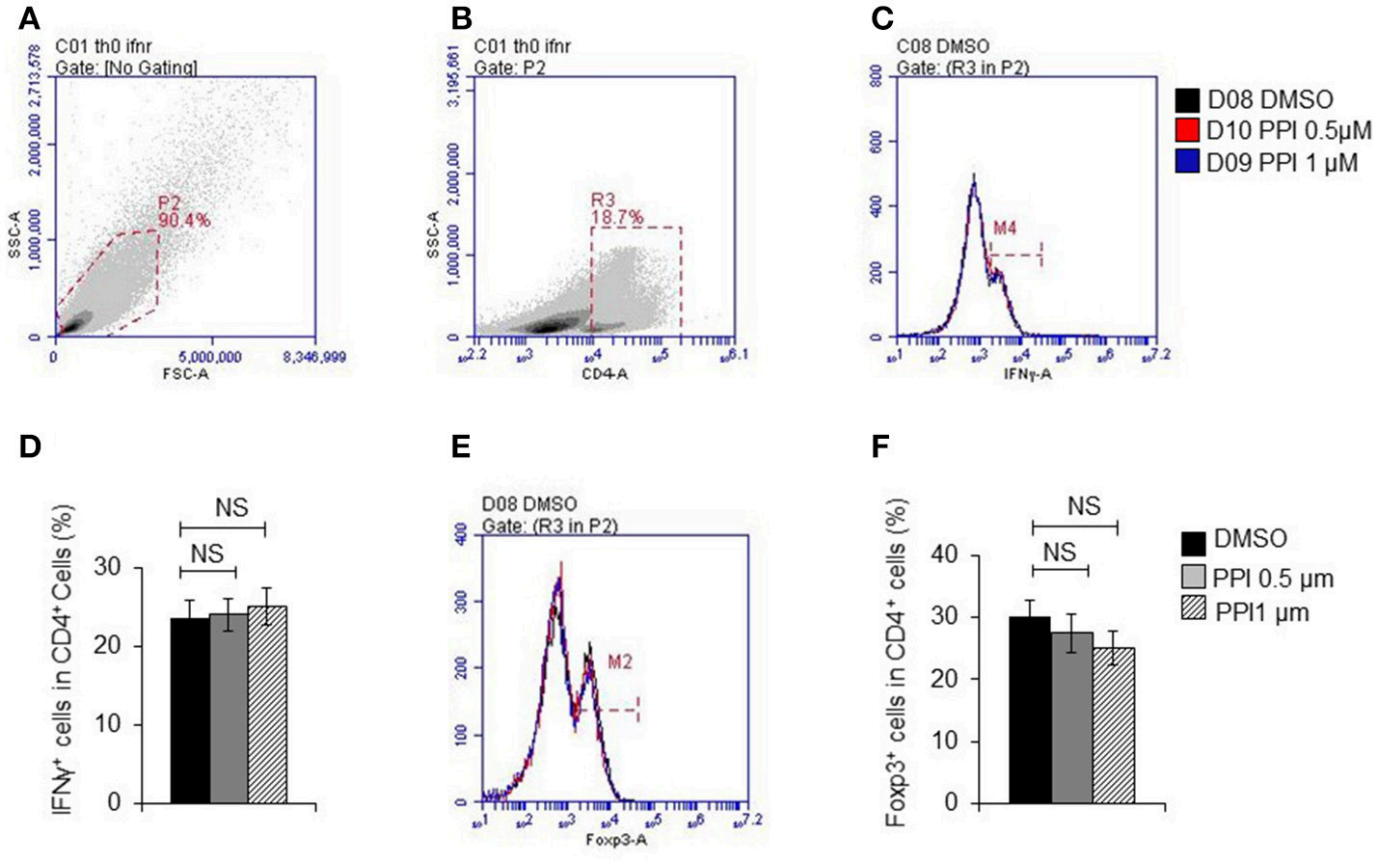
PPI did not show effect on T cell differentiation *in vitro*. **(A)** Representative images of initial gate of whole spleen cells population. **(B)** Representative images of CD4 positive cells. **(C)** IFNγ positive cells in CD4 positive cells. **(D)** Percentage of IFNγ positive cells in CD4 positive cells from 4 mice, **(E)** Foxp3 positive cells in CD4 positive cells. **(F)** Percentage of Foxp3 positive cells in CD4 positive cells from 4 mice. Data represent mean ± SEM. NS, *P* > 0.05, one-way ANOVA.

These data suggested that PPI ameliorated infiltration of inflammatory macrophages into the ankle joint of CIA mice. In order to determine whether the beneficial effect of PPI in the CIA model also comes from its action on T cells, we did immunohistochemical staining with an anti-CD3 antibody and found that CD3-positive cells were potently decreased in the PPI group compared with the Model + CMC-Na group (Figure 7J). To further confirm the effect of PPI on T cell differentiation *in vitro*, we treated T cells with PPI (0.5 and 1 μM) and did not find any effect on Th1 or Tregs differentiation (Figures 8A–F).

### PPI showed no evidence of liver or kidney toxicity

**Figure 9:**
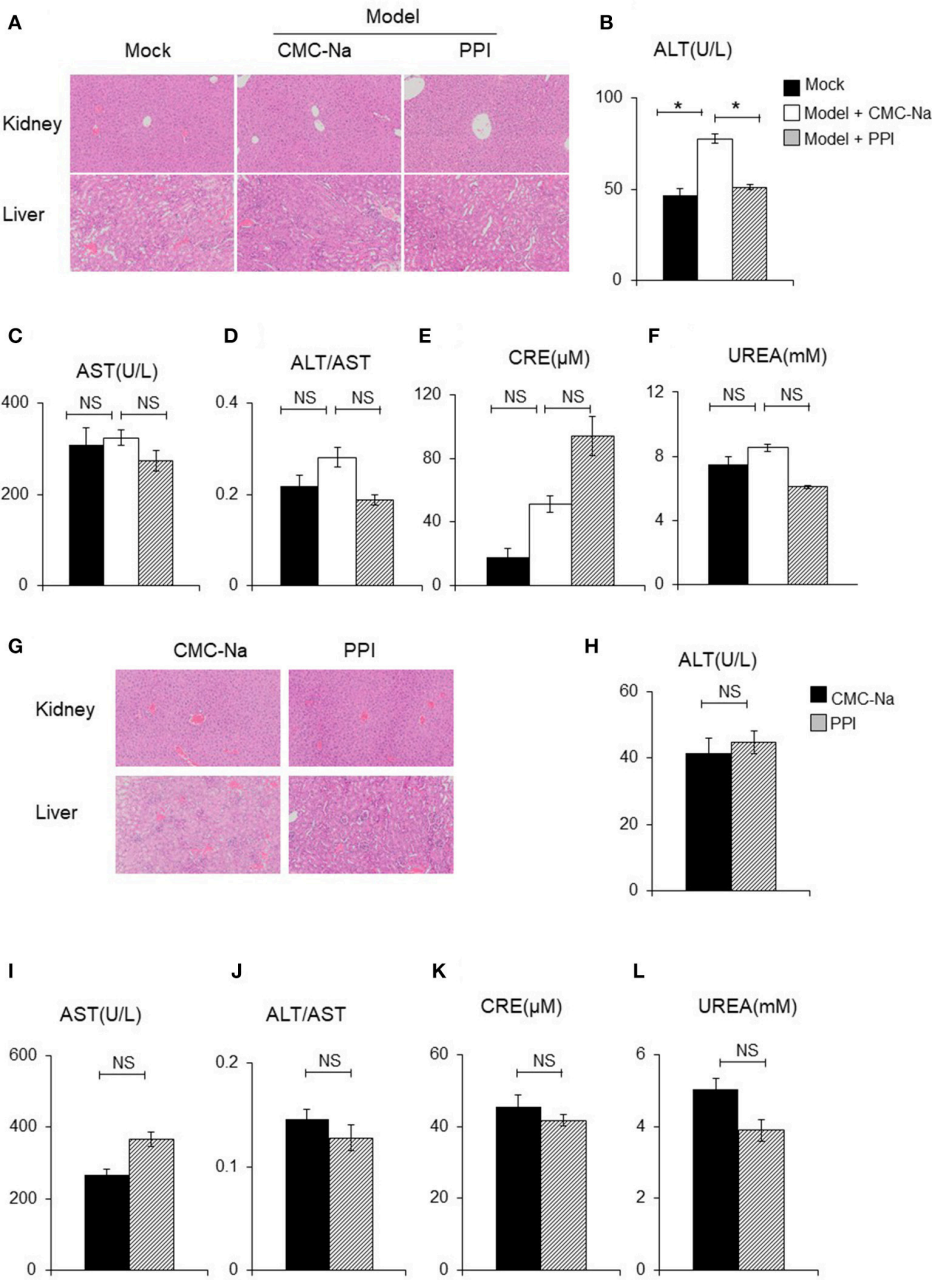
PPI did not show any damage effect on liver and kidney of CIA and normal mice. **(A)** The hematoxylin-eosin (H&E) staining of kidney and liver of CIA mice treated by CMC-Na with or without PPI (4×). **(B, C)** Serum aspartate aminotransferase (AST) and alanine aminotransferase (ALT). **(D)** The ratio of ALT/AST. **(E, F)** Serum creatinine (CRE) and blood urea nitrogen (UREA) were detected by CRE reagent kit or UREA reagent kit. **(B–F)** Data represent mean ± SEM of two pooled experiments, *n* = 4 for Mock, *n* = 7 for Model+CMC-Na and *n* = 6 for Model+PPI group. NS, *P* > 0.05, one-way ANOVA. **(G)** H&E staining of the kidney and liver of normal mice treated with CMC-Na with or without PPI for 7 weeks (4×). **(H–L)** Serum level of AST, ALT, ALT/AST, UREA, and CRE of normal mice treated by CMC-Na with or without PPI. Data represent mean ± SEM of one pooled experiments. *n* = 5 for the CMC-Na and *n* = 5 for the PPI group, NS, *P* > 0.05, *P < 0.05, Student's *t*-test.

The liver and kidney tissue, as well as peripheral blood, of Mock, Model + CMC-Na and Model +PPI group mice were obtained to examine hepatocyte/glomerulus morphological characteristics and injury in CIA mice *in vivo*. The hematoxylin and eosin (H&E) staining of the liver samples showed that the PPI group had normal hepatic lobules and hepatocytes compared to the Mock group, while the serum ALT and AST test indicated that hepatocytes in the PPI group did not show evidence of injury to that organ (Figures 9 A–D). The H&E staining of the kidney showed that the glomerulus were normal in the PPI group, and the UREA and CRE test showed that the PPI group had a normal filtration function compared with the Mock group (Figures 9A,E,F).

Next we tested whether PPI could cause hepatocyte injury or reduce glomerular filtration in the healthy mice. Ten C57/BL6 female mice were randomly divided into a normal group and PPI-treatment group by a random number table. For the PPI treatment group, PPI was intragastrically infused at a dose of 1 mg/kg once a day for 7 weeks, while the control group was administered with the carrier (CMC-Na) in the same volume. All the mice were sacrificed to harvest serum, liver, and kidney samples. Similar to the observation of CIA mice, PPI did not show any evidence of hepatocyte or glomerular injury as determined by H&E staining, as well as ALT, AST, UREA, and CRE tests (Figures 9G–L).

## Discussion

In the current study, PPI was found to attenuate NF-κB signaling in activated macrophages *in vitro* and joint inflammation of CIA mice *in vivo*.

RA is a chronic autoimmune inflammatory disease characterized by massive infiltration of diverse immune cells resulting in cartilage and bone destruction (45). In particular, numerous activated macrophages accumulate in the synovial inflammatory infiltrate and pannus cartilage interface, possess extensive pro-inflammatory, destructive, and remodeling capacities and are actively involved in the chronic inflammation and joint destruction (14, 46, 47). There are two types of macrophages, the classical activated (M1-like) and the alternatively activated (M2-like). The M1-like macrophage stimulated by LPS/IFN-γ or TNF-α/IFN-γ are featured by iNOS production, whereas the M2-like macrophage stimulated by IL-4/13 is marked by arginase expression (48). Generally, M1-like polarized macrophages are involved in resistance to pathogens while M2-like polarized macrophages help with tissue repair as well as parasite resistance, tumor growth and invasion (49). Previous studies found that in active RA, high concentrations of M1-like polarized macrophages localize in the synovium (50).

LPS is an outer membrane polysaccharide of Gram-negative bacteria, which can trigger the immune response via binding TLR4 and initiates a signaling cascade to activate the NF-κB and MAPK signaling pathways in macrophages and stimulate the production of inflammatory cytokines, chemokines and mediators (51, 52). LPS has been applied widely to accentuate or reactivate arthritis in different animal models (53–56). The effects of drug therapy on LPS-induced production of inflammatory cytokines and activated signaling pathways in macrophages are considered important for the exploration and evaluation of RA therapy (52). As it has been reported that IFN-γ is positively correlated with the severity of RA, we used LPS and IFN-γ in our study to stimulate primary bone marrow-derived macrophages and peritoneal elucidated macrophages to mimic macrophage activation in RA, and we found that PPI significantly suppressed inflammatory cytokines and mediator expression in this system, suggesting an anti-inflammatory potential of PPI.

Previously, PPI was reported to decrease the phosphorylation of the NF-κB subunit p65 and its downstream target gene expression in the hepatocellular carcinoma cell line HepG2 (27) and human osteosarcoma cells (20), and reduce protein expression of p65 in human lung cancer cells (57). And as many previous studies suggest that the blockage of NF-κB signaling pathways is a crucial strategy to control inflammatory responses of RA (12, 15–17, 58), this led us to hypothesize that PPI might have anti-inflammatory effects in RA and, if so, by inhibiting NF-κB signaling in the macrophage. Here, we found that pretreatment with PPI suppressed LPS/IFN-γ-induced phosphorylation of p-IKKα/β and p-p65, and p65 nuclear accumulation, while also inhibiting TNF-α-induced NF-κB activation. These results suggest that PPI has an inhibitory effect on NF-κB signaling in macrophages. In order to validate that the anti-inflammatory effect of PPI on LPS/IFN-γ-activated macrophages is indeed through NF-κB signaling, we used a pharmacological NF-κB signaling inhibitor, two p65 siRNAs and one p65 overexpression plasmid. To exclude the effect of PPI on other signaling pathways under LPS/IFN-γ regulation, we examined CD40 and CD86 mRNA and protein expression after LPS/IFN-γ stimulation and PPI co-treatment, and found that PPI did not decrease the production of these two markers.

On the other hand, PPI was reported to activate c-Jun expression and the JNK signaling pathway in the ovarian cancer cell line HO-8910PM (22), glioma cells (59), and human lung cancer cells (57), but we found that PPI had no effect on JNK phosphorylation. It is well known that besides NF-κB, ERK, and p38-MAPK are also important downstream mediators of LPS-TLR4 signaling and pro-inflammatory cytokines production in activated macrophage (31–35). Here, we find that PPI has no notable effect on the phosphorylation of ERK and p38-MAPK.

TLRs play a critical role in the generation of both innate and adaptive immunity and can drive inflammation. In response to LPS, the innate immune system is activated through TLRs on macrophages, resulting in the initiation and persistence of inflammation in RA (53, 60–62). After stimulation and dimerization, the TLR signaling pathways except TLR3, recruit the adaptor molecule MyD88, which recruits downstream signaling mediators, such as IRAK-1, IRAK4, and TRAF6 to form receptor complexes (63, 64). Such complexes further activate the phosphorylation of TAK1 and NF-κB, and finally the transcription of inflammatory cytokines and mediators (65). In the present study, in order to further define the mechanism of PPI action, MYD88, IRAK1, TRAF6, TAK1, and p65 on NF-κB signaling pathway was overexpressed to mimic the activation of TLR4 since the 293T cells lack of TLR4. We found that PPI inhibited NF-κB transcriptional activation when those factors above were transfected except for p65, suggesting PPI plays a role in an anti-NF-κB effect at the downstream of TAK1.

It was reported that daily intravenous injection of PPI (2.73 mg/kg body weight) for 10 days in nude mice bearing MCF-7 cells effectively decreased tumor growth without significant toxicity of the heart and liver (66). In our study, intragastric administration of PPI (1 mg/kg body weight) once a day for 7 weeks in a CIA model in DBA/1 mice attenuated joint score, synovial inflammation, bone erosion and the degree of iNOS-high macrophages in the ankle joints, without any signs of hepatocyte or glomerular injury. These results indicate that PPI effectively reduced the symptoms of synovial inflammation in the CIA mouse model without any signs of liver or kidney injury, while also suggesting PPI as a potential new drug therapy for RA.

## Conclusions

In summary, we demonstrate here for the first time, to the best of our knowledge, that PPI effectively ameliorates synovial inflammation in the ankle joint of CIA mice and suppresses pro-inflammatory mediator production of activated macrophage without any signs of liver or kidney injury *in vivo*. We also found that this compound can inhibit NF-κB signaling-mediated production of pro-inflammatory cytokines in BMMs and PEMs stimulated by LPS/IFN-γ. Together, these results suggest PPI as a new avenue for therapy of RA.

## Availability of data and material

The data supporting the conclusions of this article are included within the article.

## Author contributions

QW and XZ performed most of the experiments, analyzed the data and participated in the manuscript draft. YZ and JX helped feed the mice, finished mice treatment and the sample processing work. YL performed the inhibitor and siRNA experiment. QS, YW, and HW provided scientific input and helped with manuscript editing. QL designed the study, and drafted and finalized the manuscript. All authors read and approved the final manuscript.

### Conflict of interest statement

The authors declare that the research was conducted in the absence of any commercial or financial relationships that could be construed as a potential conflict of interest. The reviewer PT and handling Editor declared their shared affiliation at the time of review.

## Abbreviations

RA: Rheumatoid arthritis
PPI: Polyphyllin I
CIA: collagen induced arthritis
IFN-γ: interferon-γ
TNF-α: Tumor necrosis factor-α
TLRs: Toll-like receptor;IκB inhibitor of NF-κB
IKK: inhibitor of NF-κB kinase
IL: interleukin
iNOS: inducible nitric oxide synthase
IACUC: Institutional Animal Care and Use Committee
CMC-Na: sodium carboxyl methyl cellulose
BMMs: bone marrow-derived macrophages
PEMs: peritoneal elucidated macrophages
ABOG: Alcian blue-Hematoxylin-Orange G
HE: hematein eosin
AST: serum aspartate aminotransferase
ALT: serum alanine aminotransferase
UREA: blood urea nitrogen
CRE: Serum creatinine
micro-CT: Micro computed Tomography
BV: bone volume
IF: immunofluorescence
M1: macrophage classical activation
M2: alternative activation
IRAK: IL-1R associated kinase
TRAF: TNF receptor associated factor
LPS: lipopolysaccharides; nuclear factor kappa-light-chain-enhancer of activated B cells (NF-κB)
(MYD88): Myeloid differentiation primary response 88
IRAK1: interleukin-1 receptor (IL-1R) associated kinase 1
TRAF6: TNF receptor associated factors 6
TAK1: Transforming growth factor-b–activated kinase 1
FBS: fetal bovine serum
P/S: penicillin and streptomycin
M-CSF: Macrophage colony-stimulating factor; Quantitative Real-time-PCR (qPCR)
DAPI: 4',6-diamidino-2-phenylindole
BSA: Bovine Serum Albumin
SDS: sodium dodecyl sulfate
SDS-PAGE: sodium dodecyl sulfate polyacrylamide gel electrophoresis
PVDF: Polyvinylidene fluoride
TBST: tris-buffered saline and Tween 20
SEM: standard error of mean
ANOVA: Analysis of variance
ELISA: enzyme-linked immunosorbent assay
NO: nitric oxide.
